# Development and preliminary evaluation of the validity and reliability of a revised illness perception questionnaire for healthcare professionals

**DOI:** 10.1186/s12912-016-0156-4

**Published:** 2016-06-01

**Authors:** Seher Arat, Anke Van den Zegel, Maity Van Rillaer, Philip Moons, Joris Vandenberghe, Ellen De Langhe, René Westhovens

**Affiliations:** Department of Development and Regeneration, Skeletal Biology and Engineering Research Centre, KU Leuven, Herestraat 49, 3000 Leuven, Belgium; Ursulinen High School, Hoogstraat 35, 2800 Mechelen, Belgium; Midwifery Practice Leuven, Diestsesteenweg 49, 3010 Leuven, Belgium; Department of Public Health and Primary Care, KU Leuven, Kapucijnenvoer 35, Box 7001, 3000 Leuven, Belgium; Institute of Health and Care Sciences, University of Gothenburg, Gothenburg, Sweden; Department of Psychiatry, Liaisonpsychiatry, University Hospitals Leuven, Herestraat 49, 3000 Leuven, Belgium; Department of Neurosciences, KU Leuven, Herestraat 49, 3000 Leuven, Belgium; Department of Rheumatology, University Hospitals Leuven, Herestraat 49, 3000 Leuven, Belgium

**Keywords:** Validity, Reliability, Illness perceptions, Healthcare professionals, IPQ-R HP, Physical disease

## Abstract

**Background:**

Diverging perceptions between individual patients with somatic diseases and their healthcare professionals might cause problems in communication and decision-making. To date, no measurement tool is available to compare the illness perceptions between these two groups. The Revised Illness Perception Questionnaire (IPQ-R) is a validated, widely used instrument in many patient populations with somatic conditions. The aim of this study was to adapt the IPQ-R to a healthcare professional’s version (IPQ-R HP) and to perform a preliminary evaluation of its validity and reliability.

**Methods:**

After adaptation of the IPQ-R HP, 17 doctors from 3 general hospitals and 9 head nurses from a university hospital evaluated the face and content validity of the IPQ-R HP. The results were quantified using the content validity index (CVI) and a modified kappa index (k*). For the reliability measurements a group of nurses from 4 nursing wards participated at 2 time points with an interval of 4 weeks. Internal consistency and test-retest reliability were calculated.

**Results:**

Twenty-eight of the 38 items demonstrated excellent content validity and four items showed good content validity. Four items had a sufficient k* and two items had a low CVI. The average CVI of the 7 dimensions ranged from 0.66 to 0.89. The Cronbach’s alpha scores for the seven dimensions, intraclass coefficients and effect size estimates were acceptable.

**Conclusions:**

This preliminary evaluation of the IPQ-R HP shows an acceptable to good validity and reliability. Further exploration of the psychometric properties of this questionnaire in a large cohort of healthcare professionals is warranted.

**Electronic supplementary material:**

The online version of this article (doi:10.1186/s12912-016-0156-4) contains supplementary material, which is available to authorized users.

## Background

Illness perceptions are the cognitive beliefs that patients have about their condition. They refer to the cognitive depiction of an illness, reflecting how the illness is ‘pictured and stored’ in the mind [1, 2]. Illness perceptions directly influence the individual’s emotional response to the illness and guide coping behaviour such as adherence to treatment and health care use in a positive or negative way [1, 3, 4]. Over time, researchers have used various methodologies to measure the patients’ illness perceptions, ranging from questionnaires in early studies to in-depth semi-structured interviews [5].

Unfortunately, these questionnaires were not based on a generally accepted theory nor were they evaluated in different patient groups [6, 7].

Currently, the majority of studies focusing on patients’ illness perceptions are based upon Leventhal’s Self-Regulatory Model. In 1980, Leventhal and colleagues [8] developed this theoretical framework to explain why and how illness representations can differ. They identified different components including the labels, timeline, cause(s), consequences and control. This work resulted in the development of the Illness Perception Questionnaire (IPQ) that assesses these five components of illness representation. A revised version (IPQ-R) was developed in 2002 by Moss-Morris and colleagues [9]. The IPQ-R is widely used in various patient populations such as systemic sclerosis [10] rheumatoid arthritis [11, 12], psoriasis [13] and can be modified for use in a particular disease of interest. It has good psychometric properties with a good internal reliability, discrimination and predictive validity and has already been translated in different languages [9].

In daily clinical practice, the patient and the healthcare professional (HP) often have different views on the illness and its impact on a particular patient. Awareness of these divergent illness perceptions is crucial, as they can result in misunderstandings and disrupted communication when unrecognized [14–16]. Previous research evaluating the patient-professional encounter described clear differences between the perceptions of the patients with chronic obstructive pulmonary disease and asthma on the one hand and the physicians and nurse specialists on the other hand concerning timeline, control, consequences and outcomes [17]. Interestingly, a Japanese study [18] demonstrated that a gap between the patient’s and the doctor’s perceptions was the most significant predictor of doctor-shopping behaviour.

Detection of misperceptions is possible by matching a scale that assesses patients’ perceptions and perceptions of healthcare professionals (HPs). There is no appropriate and validated instrument available to measure illness perceptions of HPs caring for patients with physical diseases. At this moment questionnaires are available to measure lay perceptions of healthy people [19], illness perceptions of carers of schizophrenia patients [20] and a modified version of the illness perception questionnaire for mental health practitioners [21]. In the latter study the utility of a modified version of the IPQ was investigated to detect changes in mental health practitioners’ illness perceptions about schizophrenia after undertaking psychosocial intervention training. The modified IPQ was completed before and after the training. Afterwards the psychometric properties of the modified IPQ were tested using confirmatory factor analysis showing that a six factor model was most appropriate, but also that there was a poor fit of the items in each factor. This implied that the instrument was not valid and reliable enough to detect changes in illness perceptions.

The purpose of this study was to adapt the IPQ-R to a healthcare professional’s version and to perform a preliminary evaluation of its validity and reliability.

## Methods

The first step in the methodology was an adaptation and rewording of the IPQ-R to a healthcare professionals version. Secondly, face validity and content validity of this adapted version was evaluated in a group of physicians and head nurses. At last, the reliability measurements, i.e. the internal consistency and test-retest reliability, were assessed in a group of nurses.

### Adaptation/rewording of the IPQ-R for healthcare professionals

Four authors (SA, PM, JV and RW) discussed and agreed upon the adaptation of the 9-dimension Dutch version of the IPQ-R [22] to a healthcare professionals version (IPQ-R HP). This process comprised several rounds. An item-by-item approach was followed by a dimensional and overall evaluation. The primary goal of the process was to focus on the perception of the HP regarding the disease of a particular patient (dimensions 1, 2, 3, 4 and 6). The secondary focus of the IPQ-R HP was the view of the HP on the perceptions of that particular patient, regarding his/her illness and about its emotional impact on the patient (dimensions 5 and 7). The reformulation of the latter 2 dimensions was done in this way because these dimensions have an emphasis on the emotionality and understanding of the patient and not on the disease of the patient. We did not include the dimensions ‘illness identity’ (= perceptions of symptoms associated with the illness) and ‘causal attributions’ in the IPQ-R HP. From a HP’s perspective, these 2 dimensions are also part of illness perceptions but more related to medical knowledge or a medical judgment of the illness in comparison with patients’ illness perceptions because of their biomedical education. The other 7 dimensions of the original IPQ-R were reworded to a HP’s version. Finally, this IPQ-R HP consisted of 7 dimensions: 1) consequences (the HPs’ perception of the consequences of the illness for a particular patient); 2) timeline acute/chronic (the HPs’ perception about the illness passing quickly or not in a particular patient); 3) personal control (the HPs’ perception of the patient’s ability to control the illness); 4) treatment control (the HPs’ perception about the effectiveness of any treatment or approach to control the illness in a particular patient); 5) illness coherence (the HPs’ perception of the extent to which a particular patient understands their illness); 6) timeline cyclical (the HPs’ perception of the cyclical nature of the illness across time); and 7) emotional representations (the HPs’ perception of the patients’ emotional experience of their illness). In general, besides the rewording and reformulation, the difference between the IPQ-R and IPQ-R HP were the terms ‘I’ and ‘this patient’.

### Sampling strategy - procedure

To measure the face and content validity, a purposive sampling strategy was conducted. We had a list with the names of 20 physicians from 3 general hospitals and 11 head nurses from a large university hospital in Belgium and invited them to participate. Two sampling criteria were used to recruit the healthcare professionals: they had to be specialised in internal medicine and have active patient contact at an outpatient clinic or inpatient service. They were visited in their respective hospitals and introduced in the study (by AVdZ and MVR). Oral and written information about the study was given together with the scoring instructions and the IPQ-R HP. After one week, researchers AVdZ and MVR contacted the physicians and head nurses personally and reminded them to complete the questionnaire if needed.

For the reliability measurement, head nurses of 11 nursing wards from the university hospital were approached and asked if their nurses could participate. At these 11 nursing wards a total of 242 nurses are working. We opted for nurses to score the reliability measurements because for the face and content validity measurements already more physicians than nurses were present. Nurses received first oral information during a team meeting, after which they received written information regarding the study. They were asked to complete the IPQ-R HP on the basis of 4 patient vignettes. After an interval of 4 weeks, they were asked to complete the IPQ-R HP on the basis of 1 patient vignette that was included in the first round. The reason for reducing the number of vignettes from 4 to 1 was the indication of survey fatigue among respondents which could have an impact on the response rate. These patient vignettes were developed by SA and RW and comprised information regarding 2 patients with Systemic Lupus Erythematosus and 2 patients with Systemic Sclerosis based on real patients seen in the clinic. The information in the vignettes pertained to the patients’ clinical condition (i.e. a description of antibody profile, characteristics and complication of the disease), the medical treatment and eventual psychosocial complications and coping styles having a possible or probable impact on daily life (see Additional file 1).

### Validity

#### Face Validity

Face validity is the extent to which a test is representative for covering the concept it purports to measure at first sight [23]. The IPQ-R HP was accompanied by four questions about each dimension and also a general question at the end. These four questions were: “Are these questions a correct representation of the dimension?”; “Are the questions clear?”, “Are there questions lacking?” and “Are there redundant questions?” At last, there was an open question for further remarks. The reason why we asked, if the items per dimension are representative for a particular dimension, is because the concept of illness perceptions consists of several dimensions. So, we used the theory behind the concept of illness perceptions [8] as a backbone to rely on. In this phase, the emphasis was on the representativeness of the items covering the concept on first sight and not on removing or adding new items because of their content [24].

### Content validity

The IPQ-R HP was also tested for content validity. Content validity is the extent to which a measure represents all facets of a given construct [25]. In other words, the items on the test represent the entire range of possible items the test should cover [26, 27].

Physicians and head nurses, this was the same group as for the appraisal of the face validity, were instructed to rate the 38 items of the IPQ-R HP on a 4-point Likert scale as: “1 = not relevant”, “2 = somewhat relevant”, “3 = quite relevant”, “4 = highly relevant”. An appropriate sample size for calculating content validity ranges between 5 and 10 [26].

Based on these data, the item Content Validity Index (I-CVI) was calculated. The I-CVI is the proportion of items that received a rating of 3 or 4 by the experts. For the total instrument and each scale, a scale content validity index (S-CVI) was calculated. This is the average of all the I-CVI’s of the individual items (S-CVI_ave_). An I-CVI of 0.78 and an S-CVI_ave_ of 0.90 is considered to be excellent [27].

To counter the limitations of the CVI, each I-CVI was adjusted for chance agreement by calculating the modified kappa statistic (k*) [28]. To compute the modified kappa, the probability of chance agreement was computed first: P_c_ = [N!/A! (N − A)!] × 0.5^N^ where N is the number of experts and A is the number agreeing on good relevance (rating 3 and 4). Next, the k* was calculated with the formula k* = [I-CVI− P_c_ ]/[1− P_c_] [26]. According to the standards of Fleiss [29] and Cicchetti and Sparrow [30] the value of each k* was evaluated as poor (k <0.40), fair (k of 0.40 to 0.59), good (k of 0.60 to 0.74), or excellent (k of > 0.74).

### Reliability

To measure the internal consistency, Cronbach’s alpha values per vignette were calculated and also a total Cronbach’s alpha value was computed. Sample size estimations to accurately determine the internal consistency showed that a minimum of 17 subjects was necessary.

To evaluate the test-retest reliability, nurses were asked at time point 1 (T1) to complete the 4 vignettes and at time point 2 (T2) they were asked to complete vignette number 4 because this vignette had the best alpha values and the content was a good mix of psychosocial and clinical information. Intraclass correlation (ICC) was computed to describe how strongly illness perception dimensions in the same group resemble each other. It is a measure of the reliability of measurements. ICC can be interpreted as follows: 0–0.2 = poor agreement; 0.3-0.4 = fair agreement; 0.5-0.6 = moderate agreement; 0.7-0.8 = strong agreement; and >0.8 = almost perfect agreement. These values are arbitrary cutoffs, but similar to those used by Landis and Koch [31] for agreement of categorical items.

We also looked for differences in illness perception scores between T1 and T2 which are expressed in effect sizes. For the continuous variables, an effect size for the Wilcoxon signed rank test was calculated by $r=\frac{Z}{\sqrt{N}}$ where Z is the normal approximation of the Wilcoxon test statistic and N is the total number of participants on which Z is based. To appraise the magnitude of the effect sizes we used the cutoff values for Cohen’s r: small effect size = between 0.10 and 0.30; medium effect size = between 0.30 and 0.50 and large effect size = 0.50 or higher [32].

### Statistical analysis

Descriptive statistics were used to calculate the I-CVI values, the S-CVI_ave_ values, the P_c_ and k*. These data were analysed using Microsoft Excel (version 2011). The calculation of the Cronbach’s alpha, ICC and Wilcoxon signed rank test was carried out with SPSS version 22.0.

## Results

### Experts scoring validity

Seventeen doctors and 9 head nurses participated (response rate = 84 %). The sample of the physicians consisted of 9 men and 8 women and was composed of 4 gastroenterologists; 3 endocrinologists; 2 rheumatologists; 2 cardiologists; 2 pulmonologists; 1 nephrologist; 1 neurologist; 1 dermatologist and 1 oncologist. No further demographic data were available. The 3 doctors who did not participate were all men and gave a lack of time as a reason for not participating. The 9 head nurses, 6 women and 3 men, worked at following disciplines: cardiology; gastroenterology; rheumatology; nephrology; gynecology; ophthalmology; otorhinolaryngology and 2 pulmonology wards. One head nurse declined participation because of insecurity concerning scoring the questions correctly. The other gave no reason.

### Face validity

In Table 1, the face validity scores of the IPQ-R HP are tabulated. One of the 9 head nurses did not score the face validity questions. For almost all healthcare professionals, the questions were a correct representation of the dimensions and the questions were clear. Physicians wanted to add more questions such as items concerning self-appearance, autonomy and quality of life in the ‘Consequences’ dimension. In the ‘Timeline’ dimension they wanted to add questions regarding curability of the patient and worsening of the disease. The most redundant or overlapping questions for the physicians and head nurses were found in the dimensions Timeline acute/chronic; Personal control; Illness coherence and Emotional representations. More specifically, for the dimension ‘timeline acute/chronic’, the experts found an overlap in items 7, 8, 10 & 11. For the dimension ‘personal control’, following items were comparable for the experts: 14, 15, 17, 18. For the dimension ‘illness coherence’, the experts scored items 25, 26, 27 as comparable items. For the dimension ‘emotional representations’, items 37 & 38 were comparable.

**Table 1:** Overview appraisal face-validity of the IPQ-R HP

Dimension	Are the questions a correct representation of the dimension?		Are the questions clear?		Are there questions lacking?		Are there questions redundant?	
	Doctors *n* = 17	Nurses *n* = 8	Doctors *n* = 17	Nurses *n* = 8	Doctors *n* = 17	Nurses *n* = 8	Doctors *n* = 17	Nurses *n* = 8
Consequences	17/17	8/8	17/17	6/8	4/17	1/8	6/17	1/8
Timeline acute/chronic	16/17	8/8	15/17	8/8	4/17	0/8	7/17	4/8
Personal Control	14/17	8/8	14/17	8/8	2/17	0/8	8/17	3/8
Treatment Control	15/17	8/8	14/17	7/8	1/17	0/8	2/17	2/8
Illness Coherence	15/17	8/8	12/17	7/8	1/17	0/8	7/17	4/8
Timeline Cyclical	15/17	8/8	15/17	8/8	3/17	0/8	4/17	2/8
Emotional Representations	15/17	8/8	15/17	8/8	2/17	0/8	12/17	4/8

### Content validity

A total of 16 physicians and 9 head nurses completed the 4-point Likert scale (see Table 2). Three doctors did not complete one question or one dimension. They gave no reason why they left these items blank. This means that 12 items were rated by 15 doctors and 26 items were assessed by 16 doctors.

**Table 2:** Evaluation of the content validity of the IPQ-R HP

	Item (dimension)	Experts (*n*)	Experts with rating 3 of 4	I-CVI^a^	P_c_^b^	k*^c^	Evaluation^d^
1	The illness of my patient is serious (1)	25	23	0.92	0.0000	0.92	Excellent
2	The illness of my patient has major consequences on his/her life (1)	25	25	1.00	0.0000	1.00	Excellent
3	The illness of my patient does not have much effect on his/her life (1)	25	13	0.52	0.1550	0.43	Fair
4	The illness of my patient strongly affects the way others see him/her (1)	25	12	0.48	0.1550	0.38	Poor
5	The illness of my patient has serious financial consequences for him/her (1)	25	18	0.72	0.0143	0.72	Good
6	The illness of my patient causes difficulties for those who are close to him/her (1)	24	21	0.88	0.0001	0.87	Excellent
7	The illness of my patient will last a short time (2)	25	17	0.68	0.0322	0.67	Good
8	The illness of my patient is likely to be permanent rather than temporary (2)	25	24	0.96	0.0000	0.96	Excellent
9	The illness of my patient will last for a long time (2)	25	21	0.84	0.0004	0.84	Excellent
10	The illness of my patient will pass quickly (2)	25	8	0.32	0.0322	0.30	Poor
11	I expect that my patient will have his/her illness for the rest of his/her life (2)	24	19	0.79	0.0025	0.79	Excellent
12	The illness of my patient will improve in time (2)	25	22	0.88	0.0001	0.88	Excellent
13	My patient can do a lot to control his/her symptoms (3)	25	22	0.88	0.0001	0.88	Excellent
14	What my patient does can determine whether his/her illness gets better or worse (3)	25	21	0.84	0.0004	0.84	Excellent
15	The course of my patients’ illness depends on him/her (3)	25	21	0.84	0.0004	0.84	Excellent
16	Nothing my patient does will affect his/her illness (3)	25	19	0.76	0.0053	0.76	Excellent
17	My patient has the power to influence his/her illness (3)	25	18	0.72	0.0143	0.72	Good
18	The actions of my patient will have no effect on the outcome of his/her illness (3)	25	21	0.84	0.0004	0.84	Excellent
19	There is very little that can be done to improve the illness of my patient (4)	25	22	0.88	0.0001	0.88	Excellent
20	The treatment of my patient, will be effective in curing his/her illness (4)	25	23	0.92	0.0000	0.92	Excellent
21	The negative effects of the illness of my patient can be prevented (avoided) by his/her treatment (4)	25	23	0.92	0.0000	0.92	Excellent
22	The treatment of my patient can control his/her illness (4)	25	25	1.00	0.0000	1.00	Excellent
23	There is nothing which can help the condition of my patient (4)	25	18	0.72	0.0143	0.72	Good
24	The illness of my patient is a mystery to him/her (5)	25	23	0.92	0.0000	0.92	Excellent
25	The symptoms of the condition of my patient are puzzling to him/her (5)	25	14	0.56	0.1328	0.49	Fair
26	My patient does not understand his/her illness (5)	25	20	0.80	0.0016	0.80	Excellent
27	The illness of my patient doesn’t make any sense to him/her (5)	25	13	0.52	0.1550	0.43	Fair
28	My patient has a clear picture or understanding of his/her condition (5)	25	23	0.92	0.0000	0.92	Excellent
29	The symptoms of the illness of my patient change a great deal from day to day (6)	24	19	0.79	0.0025	0.79	Excellent
30	The symptoms of my patient come and go in cycles (6)	24	18	0.75	0.0080	0.75	Excellent
31	The illness of my patient is very unpredictable (6)	24	22	0.92	0.0000	0.92	Excellent
32	My patient goes through cycles in which his/her illness gets better and worse (6)	24	20	0.83	0.0006	0.83	Excellent
33	My patient gets depressed when he/she thinks about his/her illness (7)	24	20	0.83	0.0006	0.83	Excellent
34	When my patient thinks about his/her illness he/she gets upset (7)	24	15	0.63	0.0779	0.59	Fair
35	The illness of my patient makes him angry (7)	24	19	0.79	0.0025	0.79	Excellent
36	The illness of my patient does worry him/her (7)	24	19	0.79	0.0025	0.79	Excellent
37	Having this illness makes my patient feel anxious (7)	24	19	0.79	0.0025	0.79	Excellent
38	The illness of my patient makes him/her afraid (7)	24	19	0.79	0.0025	0.79	Excellent

^a^I-CVI (item content validity index) = number giving a rating of 3 or 4 / number experts

^b^p_c_ (probability of chance occurrence) = [N!/ A!(N-A)!] x 0,5^N^ where N = number of experts and A = number agreeing on good relevance

^c^k* = kappa designating agreement on relevance: k* = (I-CVI – p_c_) / (1 – p_c_)

^d^Evaluation criteria for k*: poor = k* < 0.40; fair = k* of 0.40-0.59; good = k* of 0.60-0.74; excellent = k* > 0.74

Twenty-eight of the 38 items had an excellent content validity (I-CVI ≥ 0.78 and k* > 0.74), 4 of the 38 items had a good content validity (I-CVI <0.78 and 0.60 ≤ k* ≤ 0.74) and 4 of the 38 items had a fair content validity (I-CVI <0.78 and 0.40 ≤ k* ≤ 0.59) (see Table 1). Two items (item 4 and item 10) had a very low modified kappa (k* < 0.40) and were considered content invalid.

The average scale content validity (S-CVI_Ave_) for each of the 7 dimensions was as follows: Consequences was 0.75; Timeline acute/chronic was 0.75; Personal control was 0.81; Treatment control was 0.89; Illness coherence was 0.74; Timeline cyclical was 0.66; and Emotional representations was 0.77. The S-CVI_Ave_ for the entire questionnaire was 0.79.

After omitting items with a fair and very low k* value the S-CVI_Ave_ for Consequences was 0.88 (without item 3 and 4), for Timeline acute/chronic was 0.83 (without item 10), Illness coherence was 0.88 (without item 25 and 27) and Emotional representations was 0.80 (without item 34). The S-CVI_Ave_ for the entire questionnaire after removing items 3, 4, 10, 25, 27 and 34 was 0.82.

For the group of the nurses, very low modified kappa values (k* < 0.40) were present for item 3 (k* = 0.26), 10 (k* = 0.09) and 27 (k* = 0.26). Item 4 had a k* value of 0.42.

### Experts scoring reliability

Four head nurses of following wards agreed with the participation of their nurses: pulmonology, rheumatology, nephrology and internal medicine. A total of 20 nurses gave consent for participation, comprising 15 women and 5 men, with a mean age of 39 years (SD = 12) and mean years of working experience of 17 years (SD = 12). Information about the non-responders is not available.

### Internal consistency

Depending on the sample size estimations, a sample of 20 nurses was large enough to adequately compute the Cronbach’s alpha. Total Cronbach’s alpha values on all vignettes rated by 20 nurses are: Consequences = 0.78; Timeline acute-chronic = 0.77; Personal control = 0.80; Treatment control = 0.50; Illness coherence = 0.75; Timeline cyclical = 0.80; Emotional representations = 0.86 (see Table 3).

**Table 3:** Cronbach’s alpha values for the four patient vignettes

Dimensions IPQ-R HP	Alpha value Vignette 1	Alpha value Vignette 2	Alpha value Vignette 3	Alpha value Vignette 4	Alpha value All vignettes
Consequences	0.77	0.81	0.73	0.83	0.78
Timeline acute-chronic	0.89	0.67	0.78	0.75	0.77
Personal control	0.80	0.88	0.77	0.76	0.80
Treatment control	0.48	0.66	0.30	0.54	0.50
Illness coherence	0.77	0.63	0.75	0.86	0.75
Timeline cyclical	0.80	0.72	0.84	0.84	0.80
Emotional representations	0.86	0.85	0.93	0.81	0.86

### Test-retest reliability

Thirteen nurses completed the IPQ-R HP on the basis of vignette 4 at T2. Table 4 displays the ICC values and Wilcoxon z-score with effect size calculation for all dimensions. The ICC values were strong for all dimensions, except for Personal control (ICC = 0.444) and Timeline cyclical (ICC = 0.417). For the latter two dimensions the ICC values can be considered as fair which means that there is a fair agreement of Personal control and Timeline cyclical at the two time points. The effect size estimates between the 2 moments was small, which means that differences in the scores of the nurses between the two time points were small.

**Table 4:** Test-retest intraclass correlation and Wilcoxon signed rank test

IPQ-R HP subscales	4 weeks test-retest intraclass correlation (95 % CI)	4 weeks test-retest Wilcoxon z score (effect size^a^)
Consequences	0.866 (0.618-0.957)	−1.221 (−0.24)
Timeline acute/chronic	0.751 (0.364-0.917)	−0.938 (−0.18)
Personal control	0.444 (−0.116-0.790)	−0.237 (−0.05)
Treatment control	0.839 (0.553-0.948)	−0.289 (−0.06)
Illness coherence	0.713 (0.264-0.908)	−1.086 (−0.21)
Timeline cyclical	0.417 (−0.148-0.777)	−0.843 (−0.17)
Emotional representations	0.844 (0.567-0.950)	−1.370 (−0.27)

^a^Effect size: small = between 0.10 and 0.30; medium = between 0.30 and 0.50

large = 0.50 or higher

## Discussion

The purpose of this study was to adapt and to perform a preliminary evaluation of the validity and reliability of the IPQ-R HP. At first sight, this IPQ-R HP has a good and acceptable face and content validity, and reliability. Experts judged the majority of the items as relevant. Item 4 and 10 were the only items with a poor or very low kappa value, indicating that these items are not valid to measure the construct, i.e. illness perceptions. Nonetheless, we decided to keep all items in the IPQ-R HP and did not omit item 4 and 10. The reason why we did not delete item 4 and 10 was that these low scores are probably due to the fact that a mix of professions, nurses and physicians, scored these items which means that they maybe gave a different meaning or interpretation to this. Only a confirmatory factor analysis can give information about items that certainly should be omitted.

The internal consistency of the 7 dimensions was acceptable and the instrument had overall good scores for the reliability measurements except for the treatment control dimension. The treatment control dimension with an alpha value of 0.50 (calculated for all vignettes) was the lowest in comparison with the other dimensions. Literature [33] states that possible reasons for a low value of alpha could be a low number of questions, poor interrelatedness between items or heterogeneous constructs. Therefore, we think that in our study a combination of a low number of items -namely, 5 items- and a low interrelatedness of these items are possible reasons why the treatment control dimension has the lowest alpha value in comparison with the other dimensions. On the other hand, experts in our study had the opinion that the items of the treatment control dimension were representative for this dimension at first sight and they also scored the content validity of this dimension as excellent. Probably a confirmatory factor analysis in a large sample of HPs can give us more insight. By comparison of the alpha values of the study of Fleming et al. [21] with the total alpha values of our study, we found that our alpha scores were higher. A possible reason is that in our study at least 4 items per dimension are present. The study of Fleming et al. [21] had 2 items per dimension. Fleming et al. [21] not only calculated Cronbach’s alpha values, but went a step further and used factor analysis to determine the underlying structure of the IPQ that they modified. The authors stated that a six factor model was the most appropriate model in comparison with a five factor model or one-dimensional model. However, no extra information was given about the p-values or correlations between the items and construct (factor) in the model. Their shortcoming was also the limited number of items (2) per factor leading to a non-representative result. A strength was the sample size of 245 mental health practitioners which was sufficient for this kind of analysis.

The strengths of our study are that we conducted this research in a group of physicians, head nurses and nurses employed in different medical disciplines and four hospitals. The sample size to measure the content validity was also much larger than previously used in similar studies [26]. The high response rate was probably due to the personal contact that we had before conducting the study. Another strength is that almost all physicians and head nurses considered the questions to be clear and providing a correct representation of the dimension at first sight. We were able to keep the original construction of the questions, which allows for matching with patients’ questionnaires at individual item level. The method of Lynn [26] is considered as an extensive method to evaluate the content validity and has shown valuable results. The results of both measurements, I-CVI and k* were in line with each other, with items not meeting the I-CVI criterion of 0.78 not showing excellent k* values and vice versa, indicating that both methods resulted in the same conclusion and were strengthening current evidence. For the reliability analyses we calculated the alpha value for each vignette separately. This gave us an idea about the amount of influence of the quality of the vignette on the reliability estimate and revealed that vignette 4 had the best alpha values.

Shortcomings of this study concerning the validity measurements, is that the use of cognitive interviewing techniques asking physicians and head nurses about their reflections concerning the individual items would, have given more background information about questions that were not clear or were skipped. Another shortcoming was that the sample size was not large enough to compute a confirmatory factor analysis because we needed then a sample between 380–570 healthcare professionals (i.e. 10–15 respondents per item) [34]. A confirmatory factor analysis would give information regarding the unidimensionality of the subscales and also provides information about the relationship between each item and the subscale. For the reliability measurements, it was difficult to motivate the nurses to complete the 4 vignettes and it was even much more difficult to motivate them to complete one vignette for a second time. Therefore, we used vignette 4 for the retest, which was a good mix of clinical and psychosocial information and had also the highest alpha value. This could have led to an overestimation of the intraclass coefficient because we used the vignette with the highest interrelatedness of items. Reasons for the low response rate were no time, too many questionnaires and too difficult vignettes. For planning further research, the number of the vignettes have to be taken into consideration.

Another limitation was that the reliability estimates are based just on a sample of nurses. It is unclear whether these results are generalizable to physicians because nurses and physicians differ in a variety of aspects like education, patient contact, responsibility for diagnostics and treatment. As a last point to consider we want to mention that in the adaptation process of the IPQ-R to the IPQ-R HP we omitted the identity and causality dimensions of illness representations. Our reasoning was that these 2 dimensions are –in comparison with patients’ perceptions- more related with biomedical knowledge. A remark on this is on the one hand, that treatment decisions are often based on physicians’ representations of the identity and causal attributions dimensions and on the other hand it is possible that conflicts between patients and physicians arise when they differ in their opinions about which symptoms relate to a specific illness or which factors caused a particular disease.

After a comprehensive validation process, we can explore the potential applications of this questionnaire in patient care. This tool is useful for investigating the causes of misunderstandings and conflicts that have arisen between medical staff and patients. When differences in perceptions between patients and HPs are detected than these differences can be discussed using this tool by comparing the patient’s and HP’s version with each other. In this way, HPs can reflect upon their own beliefs and how much it differs from patients’ beliefs. When HPs are aware of these differences they can work in a patient-centered manner during patient education sessions which means that some items or some dimensions can be a stepping stone to tailor information for a particular patient. With the IPQ-R HP areas of disagreement between patients’ and HPs’ perceptions can be pinpointed in a more detailed way which is an advantage because in this way the communication and shared understanding between HPs and patients can be enhanced. This is important because doctor–patient communication is a powerful indicator to achieve quality in care determining patients’ self-management behaviour and ultimately health outcomes [35, 36].

Practically, the patient can complete their version in the waiting room- this means before the doctor has seen him/her- and the doctor or other HP can complete the IPQ-R HP after the patient’s visit. These questionnaires can be completed in every setting, i.e. an inpatient or outpatient setting. It is important that this happens when the HP has formed an idea about the patient’s physical and mental condition. The next stage is the comparison of these 2 instruments which can be done easily by the HP. We do not think it is useful to complete this questionnaire each moment the patient encounters an HP. The completion of these instruments can have an added value especially at diagnosis and when there is a flare or acute exacerbation of the patient’s condition because illness perceptions are relatively stable but may show some fluctuations at those time points [37].

## Conclusion

The IPQ-R HP appears adequate and useful to assess the perception of healthcare professionals concerning the illness of an individual patient and produces -in this preliminary phase- reliable and valid output. A more extensive validation process is needed in a large cohort of healthcare professionals to explore the psychometric properties of this questionnaire prior to a widespread use in clinical practice. Moreover, a large cohort of healthcare professionals is needed to investigate the factor structure of the IPQ-R HP with the aim to determine which of the items best represent each of the illness perception dimensions. In this way it is possible to have more insight in the construct validity of the IPQ-R HP.

## Abbreviations

HP, healthcare professional; HPs, healthcare professionals; ICC, intraclass correlation; I-CVI, item content validity index; IPQ, illness perception questionnaire; IPQ-R HP, revised illness perception questionnaire for healthcare professionals; IPQ-R, revised illness perception questionnaire; S-CVI, scale content validity index; S-CVI_Ave_, average scale content validity index

## Additional file

Additional file 1: Overall description of the four patient vignettes. (DOCX 12 kb)
