# Supplementary material for: Development and preliminary evaluation of the validity and reliability of a revised illness perception questionnaire for healthcare professionals
Source: BMC Nurs. 2016 Jun 1;15:34. doi: 10.1186/s12912-016-0156-4 (PMC4888540; doi:10.1186/s12912-016-0156-4)
Supplement: Additional file 1: — Overall description of the four patient vignettes. (DOCX 12 kb) [file 12912_2016_156_MOESM1_ESM.docx]

**Description of the four patient vignettes**

-The **first vignette** describes a 28-year old Systemic Lupus Erythematosus (SLE) patient with a positive auto-antibody profile, proteinuria and lupus nephritis. She develops an epileptic attack and medication is started by her treating physician for the epileptic attack and lupus nephritis. Hereafter, her condition stabilizes. The patient herself wants a dose reduction of the anti-epileptic medication but is also afraid for a new epileptic attack after the dose reduction.

-The **second vignette** describes a 38-year old man who develops diffuse Systemic Sclerosis (SSc) with organ involvement more specifically the heart, lungs and muscles are involved. He has also a more progressive skin fibrosis due to his condition. Medication to stop disease progression is started but his physical condition deteriorates. The organ involvement spreads with chronic diarrhea and erectile dysfunction as a result. After intravenous infusions of a biological medication, disease activity stabilizes and he can fulfill his daily activities. Currently, he works part-time in an administrative function. His functionality and coping with his chronic condition improved.

-The **third vignette** is about a 35-year old women, diagnosed with SLE, and who develops 9 years after diagnosis, lupus nephritis. Medication has been started but the renal function deteriorates. This patient has a relationship and an 8-year old son. She works fulltime as a shop assistant. At the last consultation she had no complaints due to her disease except some fatigue. Her medication was switched because of a pregnancy wish.

-The **fourth vignette** is concerning a 42-year old patient diagnosed with diffuse SSc and interstitial lung disease. A year after diagnosis, she complains of dyspnea, coughing and itching. The coughing is very disturbing because of social isolation. Two years after the diagnosis of SSc her lung function deteriorates. After several intravenous infusions of a biological medicine, her disease activity does not improve and her treating physicians decide to conduct autologous stem cell transplantation. Hereafter, her quality of life improves but she finds it difficult to cope with the uncertainty of her future and the unpredictability of her disease. At this moment, she has no relationship but gets most of the emotional social support from her brothers and friends.
